# Antibacterial activity of *Dioscorea bulbifera* Linn. extract and its active component flavanthrinin against skin-associated bacteria

**DOI:** 10.1186/s12906-024-04480-8

**Published:** 2024-05-02

**Authors:** Donruedee Sanguansermsri, Phanchana Sanguansermsri, Kittisak Buaban, Vachira Choommongkol, Chareeporn Akekawatchai, Noree Charoensri, Ian Fraser, Nalin Wongkattiya

**Affiliations:** 1 Department of Microbiology and Parasitology, Faculty of Medical Science, Naresuan University, Phitsanulok, 65000 Thailand; 2 Center of Excellence in Medical Biotechnology, Faculty of Medical Science, Naresuan University, Phitsanulok, 65000 Thailand; 3 Department of Biochemistry, Faculty of Medical Science, Naresuan University, Phitsanulok, 65000 Thailand; 4 Program in Biotechnology, Faculty of Science, Maejo University, Chiang Mai, 50290 Thailand; 5 Department of Chemistry, Faculty of Science, Maejo University, Chiang Mai, 50290 Thailand; 6 The Center of Excellence in Agricultural Innovation for Graduate Entrepreneur (AgrInno), Maejo University, Chiang Mai, 50290 Thailand; 7 Department of Medical Technology, Faculty of Allied Health Sciences, Thammasat University, Pathumthani, 12121 Thailand; 8 Department of Medical Technology, Chiangmai Neurological Hospital, Chiangmai, 50200 Thailand; 9 School of Chemistry, Monash University, Clayton, VIC 3800 Australia

**Keywords:** *Dioscorea bulbifera* L., Flavanthrinin, Antibacterial activity, Skin-associated bacteria

## Abstract

**Background:**

*Dioscorea bulbifera* Linn. has been used for wound care in Thailand. However, a comprehensive evaluation of its antibacterial activity is required. This study aimed to investigate the antibacterial efficacy of *D. bulbifera* extract against skin-associated bacteria and isolate and characterize its active antibacterial agent, flavanthrinin.

**Methods:**

Air-dried bulbils of *D. bulbifera* were pulverised and extracted with hexane, dichloromethane, ethyl acetate, methanol, ethanol, and distilled water; vacuum filtered; concentrated; freeze-dried; and stored at -20 ºC. Antibacterial activity of the extracts was assessed using microdilution techniques against several skin-associated bacteria. Thin-layer chromatography (TLC) bioautography was used to identify the active compounds in the extract, which were fractionated by column chromatography and purified by preparative TLC. The chemical structures of the purified compounds were analysed using nuclear magnetic resonance (NMR). The cytotoxicity of the extract and its active compounds was evaluated in Vero cells.

**Results:**

The ethyl acetate extract exhibited distinct inhibition zones against bacteria compared to other extracts. Therefore, the ethyl acetate extract of *D. bulbifera* in the ethyl acetate layer was used for subsequent analyses. *D. bulbifera* extract exhibited antibacterial activity, with minimum inhibitory concentrations (MICs) of 0.78–1.56 mg/mL. An active compound, identified through TLC-bioautography, demonstrated enhanced antibacterial activity, with MICs of 0.02–0.78 mg/mL. NMR analysis identified this bioactive compound as flavanthrinin. Both *D. bulbifera* extract and flavanthrinin-containing fraction demonstrated potent antibacterial activity against *Staphylococcus aureus*, methicillin-resistant *S. aureus* (MRSA), and *S. epidermidis*. The flavanthrinin containing fraction demonstrated low cytotoxicity against Vero cells, showing CC_50_ values of 0.41 ± 0.03 mg/mL. These values are lower than the MIC value, indicating that this fraction is safer than the initial ethyl acetate extract.

**Conclusions:**

*Dioscorea bulbifera* extract and its bioactive component flavanthrinin demonstrated significant antibacterial activity against the skin-associated bacteria Staphylococci, including MRSA. Flavanthrinin has potential as a complementary therapeutic agent for managing skin infections owing to its potent antibacterial effects and low cytotoxicity.

## Background

Wounds are a common health issue that can lead to loss of the epithelium and underlying connective tissue. They can be caused by various physical, chemical, thermal, microbial, and immunological factors. Skin infections and wounds require special attention, as they increase the risk of bacterial, fungal, and viral infections [1, 2]. Microbial species associated with wound infections include *Staphylococcus aureus*, *Streptococcus* spp., *Escherichia coli*, *Pseudomonas aeruginosa*, *Proteus* spp., *Klebsiella* spp., *Enterobacter* spp., and *Candida* spp. [3]. In particular, the prevalence and virulence of *Staphylococcus aureus* have raised concerns owing to its high frequency of association with wound infections and its ability to induce various complications [4]. These pathogens can seriously delay the wound healing process by disrupting normal clotting mechanisms, promoting disordered leukocyte function and poor-quality granulation tissue formation, reducing the tensile strength of connective tissue, and impairing epithelialisation. Although effective, modern antibiotics and treatments may present undesirable side effects such as gastrointestinal disturbances (e.g., diarrhea and nausea), allergic reactions, and, critically, the development of antibiotic resistance. These concerns necessitate the exploration of alternative antimicrobial sources, such as medicinal plants, which may offer effective treatments with potentially fewer adverse effects.

*Dioscorea bulbifera* Linn. (*D. bulbifera*), commonly known as air potato, originated in Africa and Southern Asia. It is grown in many regions of Thailand where it is known as ‘Wan Phra Chim’. *D. bulbifera*, as an important medicinal plant, is used in traditional Indian and Chinese medicines to treat various diseases. Traditional Chinese medicine has long been used to treat haemoptysis, epistaxis, pharyngitis, goitre, pyogenic skin infections, scrofula, trauma, orchitis, and cancer. *D. bulbifera* is used to treat thyroid diseases and cancers in China [5, 6]. In Bangladesh, it is used to treat cancer and leprosy [7]. This plant has been studied pharmacologically for its anti-HIV, anti-inflammatory, antioxidant, antimicrobial, antiviral, antifungal, and anthelmintic properties [8]. These remarkable characteristics have made it a mainstay in traditional Indian, Chinese, and Thai medicines for the treatment of various diseases. While *Dioscorea bulbifera* has been recognized for its wound healing, anti-inflammatory, and antioxidant properties, aligning with its traditional usage across various cultures, there are indeed reports of its antibacterial activities. However, the scientific literature lacks detailed identification of the specific compounds within *D. bulbifera* that are responsible for these antibacterial effects, especially against skin-associated bacteria [9–11].

A recent clinical study conducted across 18 government hospitals in Thailand demonstrated the efficacy of *D. bulbifera* in significantly reducing wound size, further underscoring its therapeutic potential. A high percentage of patients expressed willingness to continue using *D. bulbifera* as a wound treatment, highlighting its potential as a cost-effective wound care strategy, particularly in resource-limited settings [12]. Despite promising results of its clinical use in Thailand, the safety and efficacy of *D. bulbifera* Linn. in bacterial infection-associated wound healing is not fully understood. Therefore, the present study aimed to investigate its antibacterial activity and identify the active components of *D. bulbifera* against common pathogens associated with skin and wound infections. To achieve this, antimicrobial activity assay and thin-layer chromatography (TLC) bioautography were conducted to identify the active constituents. Subsequently, the active components were fractionated using column chromatography and purified via preparative TLC. Nuclear magnetic resonance (NMR) analysis was performed to elucidate the chemical composition of the bioactive compounds in *D. bulbifera*. The results of this study contribute to the development of alternative and effective antimicrobial sources for the treatment of skin infections and wounds.

## Materials and methods

### Plant material

**Fig. 1:**
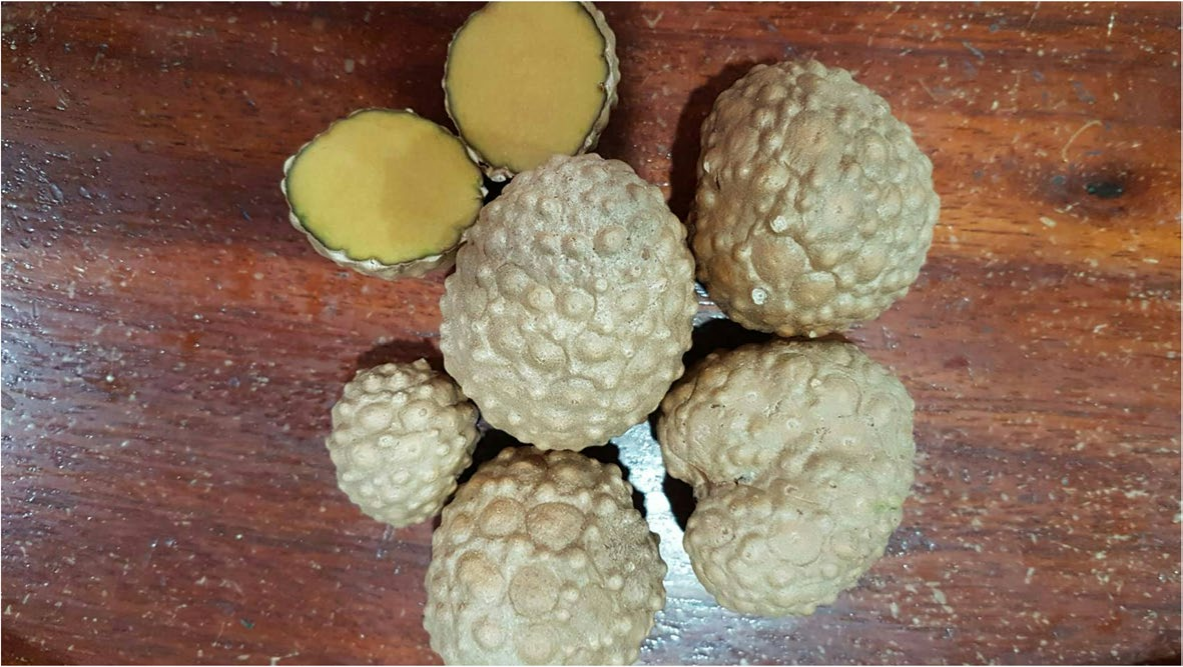
The bulbils of *Dioscorea bulbifera* Linn. collected from Suphanburi, Thailand

The bulbils of *D. bulbifera* were collected from Suphanburi, Thailand (Fig. 1). The plant was authenticated at the Faculty of Science, Maejo University, Chiang Mai, Thailand and assigned specimen voucher number MJU 21–007.

### Extraction and isolation

Air-dried bulbils of *D. bulbifera* were pulverised and extracted thrice (72 h each) with hexane, dichloromethane, ethyl acetate, methanol, ethanol, and distilled water. The extract was filtered through Whatman No. 1 filter paper under vacuum and concentrated by removing the solvent using a rotary evaporator. The extract was freeze-dried and stored at -20 ºC prior to use.

### Bacterial strains and culture conditions

Common skin pathogens were selected to evaluate the antimicrobial activities of *D. bulbifera* extracts. The bacterial strains used were *Staphylococcus aureus* DMST 8840, methicillin-resistant *Staphylococcus aureus* (MRSA) DMST 20651, *Staphylococcus epidermidis* DMST 3547, *Staphylococcus epidermidis* DMST 4343, and *Pseudomonas aeruginosa* DMST 4739. Bacteria were obtained from the Department of Medical Science of Thailand (DMST), Ministry of Public Health. They were sub-cultured on brain heart infusion agar (BHA) and incubated at 37 °C for 24 h before testing.

### Determination of Minimum inhibitory concentration (MIC)

The modified MIC was determined as described previously [13]. Briefly, 64 mg/mL extract was prepared using 10% DMSO diluted in Mueller Hinton broth (MHB) and twofold diluted in the range of 0.006–64 mg/mL with Mueller Hinton broth (MHB) in a 96-well microtiter plate. DMSO (10%) was used as the control. Fifty microlitres of the extract and control were analysed. The tested bacterial strains were adjusted to a McFarland standard turbidity of 0.5, followed by 1:100 dilution in MHB. A bacterial suspension (50 µL) was added to each well. Microtiter plates were tapped at the four corners to ensure a well-mixed solution. Tetracycline was used as the positive control. The plates were incubated at 37 °C for 24 h. Each MIC determinations was performed in triplicates. MIC was defined as the lowest concentration of the extract that showed no visible bacterial growth.

### Active compound isolation

#### TLC and TLC-bioautography

The active compounds in the extract that inhibited the tested bacteria were identified using TLC-bioautography. The procedures were performed as previously described [14]. Two Silica Gel plates (Silica Gel 60 F254; Merck, Germany) were prepared and subjected to identical conditions. The extract was separated using chloroform:methanol:water (8:2:0.2) as mobile phase. Separation was performed in a saturated chamber. The TLC plates were dried in a fume hood for 15 min to remove the solvent. The compounds separated on the first TLC plate were either visualised under a UV lamp (254 nm; Upland, CA, USA) or sprayed with acetic acid:sulfuric acid:anisaldehyde (98:1:1) solution. The spots were marked with a pencil and the retention values (R_f_) were measured. The second TLC plate was overlaid with molten BHA seeded with the bacteria (0.5 McFarland standard turbidity). The TLC plates were incubated in a moist chamber at 37 °C for 24 h and sprayed with 1% thiazolyl blue tetrazolium bromide (AppliChem, Spain) prior to incubation at room temperature for 10 min. Clear spots appearing on a purple background suggested the presence of an antibacterial agent. The second TLC plate was compared in parallel with the first plate; spots on the first plate with R_f_ values corresponding to those in the antibacterial zone revealed the active antibacterial component.

#### Purification technique using column chromatography

Silica gel 60 PF254 (Kieselgel 60, 70–320 mesh; Merck) was employed column chromatography to isolate the target compound. An isocratic elution method was used for the first round of column chromatography, wherein a mobile phase composed of ethyl acetate (5% to 100% in hexane) and methanol (5% to 100% in ethyl acetate) was employed. Fractions were collected based on the colours of the elutions. The fractions were then subjected to TLC-bioautography to determine their chemical and active antibacterial compositions.

Fractions exhibiting antibacterial activity were subjected to a second round of column chromatography using the same stationary phase and ethyl acetate, ranging from 5 to 100% in hexane, to isolate the individual chemical components. To determine the active antibacterial composition, the fractions from the second round of column chromatography were subjected to TLC.

#### Purification technique using preparative TLC (PTLC)

The fraction containing the active antibacterial compounds was purified and separated using silica gel 60 F254 plates (1.5 mm thickness; Sigma-Aldrich, MA, USA). A mobile phase composed of dichloromethane:ethyl acetate:hexane (5:20:75) was used for the chromatographic separation. Fractions exhibiting antibacterial activity were collected by scraping with a scraper and extracted with ethyl acetate. The resulting solution was filtered through 0.45 filter (Labfil, China). The purified samples were subjected to vacuum desiccation to eliminate residual moisture. The sample was subsequently dissolved in acetone and transferred to an NMR sample tube for further structural analysis using NMR spectroscopy.

### NMR analysis

The chemical structures of the purified samples were analysed using ^1^H-NMR and ^13^C-NMR spectroscopy. NMR spectra were acquired using a Bruker DRX 400 MHz spectrometer (Bruker, MA, USA), and chemical shifts were reported in parts per million (ppm) downfield of tetramethylsilane (TMS). The spectra were obtained in acetone, with the chemical shifts referenced to the residual acetone peak (1H: δ 22.84, 13C: δ 206.26). The coupling constants (J values) are reported in hertz (Hz). The multiplicity of peaks is indicated by the following abbreviations: s (singlet), d (doublet), dd (doublet of doublets), and m (multiplet).

### Cytotoxicity assay

Vero cells (African green monkey kidney cells) were grown in a monolayer of Dulbecco's modified Eagle’s medium supplemented with 5% foetal bovine serum. The MTT assay was used to evaluate cytotoxicity. Briefly, 96-well plates were seeded with 100 mL of Vero cells at a density of 2.5 × 10^5^ cells/mL and propagated at 37 ºC with 5% CO_2_ for 1 d. Fresh maintenance medium containing concentrations from 0.002 to 2.690 mg/mL of the ethyl acetate extract and subfractions was added to the wells. The cells were incubated at 37 ºC with 5% CO_2_ for 1 h before adding 20 mL of MTT reagent to each well and incubating for additional 4 h. Thereafter, the formazan crystals were solubilised using diluted HCl (0.04 mol/L) in isopropanol, and the absorbance was determined at 490 nm using a Biotek ELISA plate reader (Agilent, CA, USA) with a reference wavelength of 620 nm. The 50% cytotoxic concentration (CC_50_) was calculated as the concentration that caused visible morphological changes in 50% of Vero cells [15].

## Results and discussion

### Antibacterial activity

The inhibitory effects of *D. bulbifera* crude ethyl acetate extract and antibacterial fraction on all tested bacteria were demonstrated by the MIC, as presented in Table 1. The MIC values ranged from 0.78 to 1.56 mg/mL and 0.02 to 0.78 mg/mL for the ethyl acetate extract and antibacterial fraction, respectively. The strongest antibacterial effect of the *D. bulbifera* fraction was observed against *S. epidermidis* 3547. The *D. bulbifera* ethyl acetate extract demonstrated considerable antibacterial activity, as indicated by its MIC values. The antibacterial fraction exhibited significantly stronger activity against the tested bacterial strains, with lower MICs across all species. This suggests that a potential concentration of active constituents within this fraction amplifies its antimicrobial effect.

**Table 1:** Minimum inhibitory concentrations (MIC) of ethyl acetate extract and flavanthrinin-containing fraction

Sample	MIC (mg/mL)				
	*S. aureus* DMST 8840	MRSA DMST 20651	*S. epidermidis* DMST 3547	*S. epidermidis* DMST 4343	*P. aeruginosa* DMST 4739
Ethyl acetate extract	0.78	1.56	1.56	0.78	1.56
Flavanthrinin-containing fraction	0.04	0.04	0.02	0.04	0.78
Tetracycline	0.015	0.03	0.015	0.12	0.06

The antimicrobial activity of *D. bulbifera* against various bacteria has been reported previously. The methanol extract of *D. bulbifera* bulbils exhibited antimicrobial activities against mycobacteria and multidrug-resistant gram-negative bacteria, such as *E. coli*, *Enterobacter aerogenes*, *Klebsiella pneumoniae*, *P. aeruginosa*, *Mycobacterium smegmatis*, and *Mycobacterium tuberculosis*. Plants of the genus Dioscorea, such as *Dioscorea deltoidea*, have also demonstrated inhibitory effects against *S. aureus*, *P. aeruginosa*, and *E. coli* [16]. *D. bulbifera* exhibits strong and broad-spectrum antibacterial effects against various microorganisms, making it a potential effective therapeutic against bacterial infections, including multidrug-resistant strains. Compounds isolated from *D. bulbifera*, such as bafoudiosbulbin A and B, 8-epidiosbulbin E acetate, vanillic acid, isovanillic acid, and dihydrodioscorine, have demonstrated potent antibacterial activity. Diosgenin, a compound found in *D. bulbifera*, effectively inhibits the growth of both Gram-positive bacteria (including *Bacillus subtilis*, *Bacillus cereus*, *Staphylococcus aureus*, and *Staphylococcus epidermidis*) and Gram-negative bacteria such as *E. coli* and *Salmonella typhi* [7]. In addition, the aqueous extracts of *D. bulbifera* displayed significant antibacterial activity against *E. coli*, whereas its ethanolic extracts were notably potent against *C. albicans* and S*. aureus* [16].

### Identification and efficacy of active compounds

**Fig. 2:**
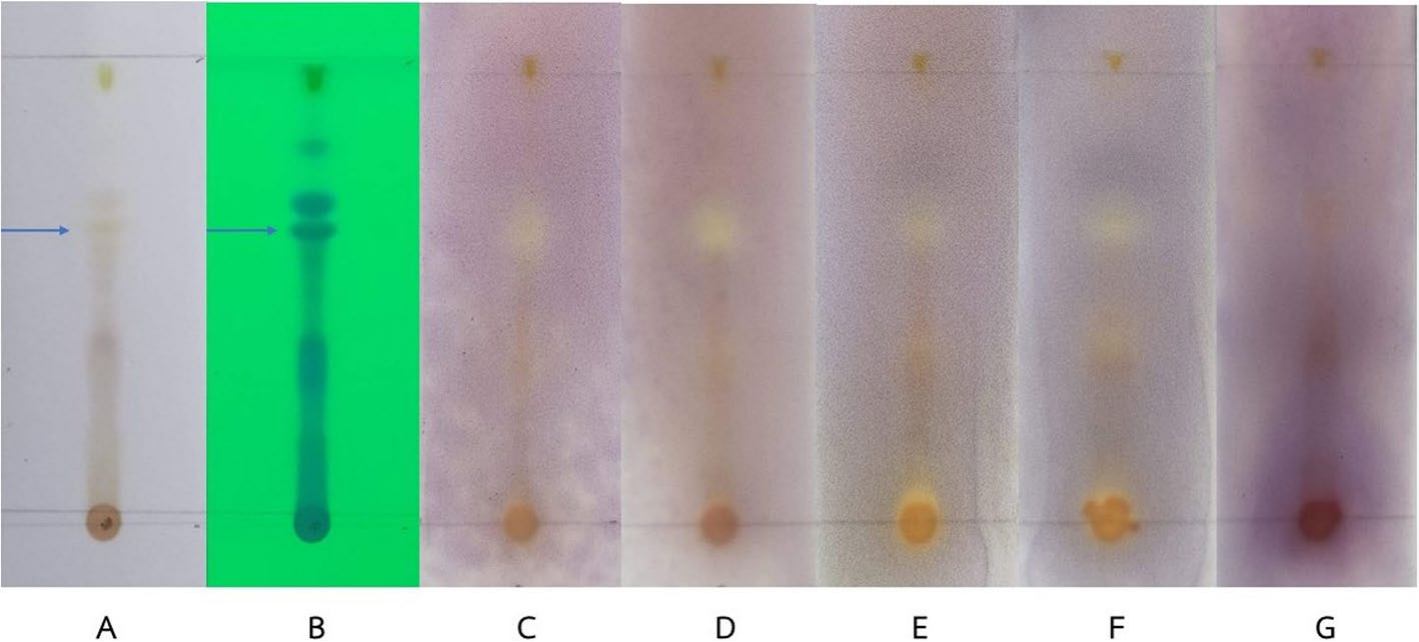
Chromatographic analysis and bioautography of *D. bulbifera* ethyl acetate extract extract to evaluate its antibacterial activity against *S. aureus*, MRSA, *S. epidermidis*, *S. pyogenes*, and *P. aeruginosa*. TLC ethyl acetate extract: visible light (**A**), under UV 254 nm (**B**), bioautography; *S. aureus* 8840 (**C**), MRSA 20651 (**D**), *S. epidermidis* 3547 (**E**), *S. epidermidis* 4343 (**F**), and *P. aeruginosa* 4739 (**G**)

The chemical components and antibacterial activity of the ethyl acetate extract of *D. bulbifera* were investigated using TLC-bioautography with six different solvent systems: hexane, dichloromethane, ethyl acetate, ethanol, methanol, and water. The results showed that the ethyl acetate extract exhibited distinct inhibition zones against bacteria compared to the methanol and ethanol extracts which only showed inhibition zones at the spot of the extract drop (not shown). Therefore, the ethyl acetate extract of *D. bulbifera* was used to assess the antibacterial activity and purified by column chromatography to isolate the antibacterial compounds. Figure 2 shows that a compound in the ethyl acetate extract (indicated by the arrow) exhibited antibacterial activity against *S. aureus*, MRSA, *S. epidermidis*, and *S. pyogenes* (lanes C–F). However, no activity was observed against the gram-negative bacterium *P. aeruginosa* 4739 (lane G). These findings suggest that the ethyl acetate extract of *D. bulbifera* contains potential antibacterial compounds that should be further investigated for therapeutic applications. However, the difference in antibacterial activity between TLC bioautography and Table 1 results, especially against *P. aeruginosa*, is likely due to concentration changes post-TLC separation. Such separation might not fully represent the synergistic antibacterial effects seen with the crude mixture. TLC bioautography, which highlights individual compounds, might not fully demonstrate the collective antibacterial strength of the extract.

The resistance of *P. aeruginosa* against the ethyl acetate extract of *D. bulbifera* in the present study may be due to its innate defence mechanisms, such as the presence of an outer membrane and efflux pumps, which can decrease its susceptibility to antibacterial agents. In contrast, a previous study using *D. bulbifera* methanol extract, fractions, and compounds demonstrated promising results against multiple drug-resistant bacteria, including *P. aeruginosa* [10]. This discrepancy in outcomes could be attributed to the different compounds extracted using methanol. These compounds may possess broad-spectrum antibacterial activity or be capable of overcoming resistance mechanisms in *P. aeruginosa*. The differences in solvent polarity between methanol and ethyl acetate likely account for the observed variation in antibacterial efficacy. The higher polarity of methanol enables the extraction of a more diverse set of bioactive compounds, potentially conferring effectiveness against *P. aeruginosa*. Previous studies have identified certain diterpenoids (bafoudiosbulbins and 2,7-dihydroxy-4-methoxyphenanthrene) as pivotal for enhanced antibacterial activity, and their unique structural attributes may bolster their antibacterial potency [10].

**Fig. 3:**
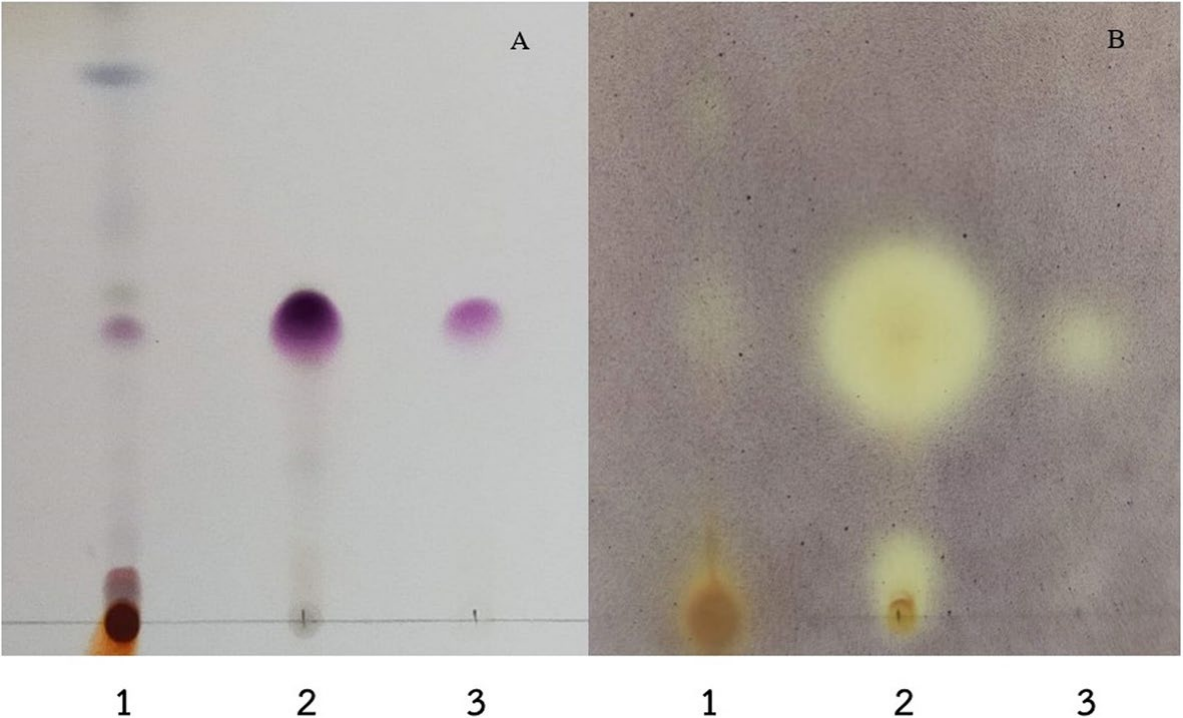
Thin layer chromatography (TLC) (**A**) and bioautography using *S. aureus* DMST 8840 (**B**); ethyl acetate extract (1), antibacterial fraction (2), active antibacterial component from PTLC (3)

The ethyl acetate extract of *D. bulbifera* was purified in a three-step process using ethyl acetate. The first two steps involved column chromatography, followed by final purification by PTLC. The fractions collected in the first step were tested for antibacterial activity, and fractions 13 and 14 exhibited such activity. These two fractions were subjected to a second round of column chromatography using the same mobile phase. Antibacterial activity was detected in subfractions 11–17 collected during this step, which were further purified by PTLC. TLC-bioautography of ethyl acetate extract, antibacterial fraction, and active antibacterial components from PTLC was performed against *S*. *aureus* DMST 8840 (representative of all tested Staphylococci) (Fig. 3).

### NMR analysis of the antibacterial compound

**Fig. 4:**
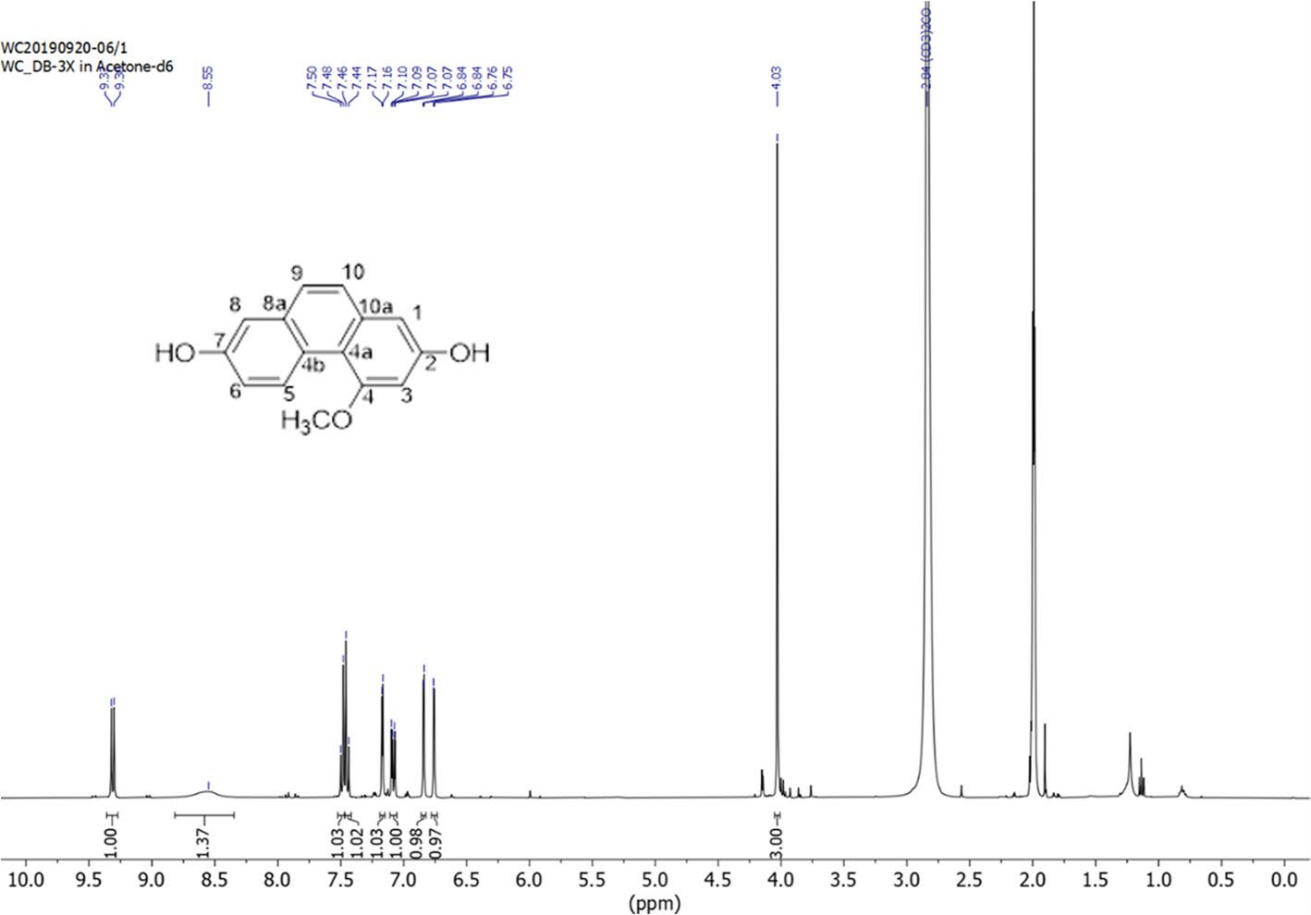
Structure of flavanthrinin elucidated from ^1^H-NMR spectrum

The antibacterial compound isolated from the *D. bulbifera* extract was subjected to 1H-NMR spectroscopy to elucidate its chemical structure. The resulting ^1^H-NMR spectrum, indicative of the pure compound's structure, is depicted in Fig. 4. This spectral analysis confirms the identity of the compound as flavanthrinin. A comparison between the ^1^H-NMR data of the bioactive compound isolated in the present study and flavanthrinin previously reported in the orchids *Bulbophyllum reptans* and *B. vaginatum* [17, 18] revealed similar antibacterial activities (Table 2). This similarity suggests that the bioactive antibacterial compound obtained from *D. bulbifera* is flavanthrinin. This finding was consistent with the results of TLC-bioautography, which identified flavanthrinin as an active compound against *S. aureus*, MRSA, and *S. epidermidis*.

**Table 2:** Comparison of ^1^H-NMR spectra of the separated bioactive compound and flavanthrinin

Position	Biologically active compound separated from *Dioscorea bulbifera* L	Flavanthrinin (2,7-dihydroxy-4-methoxyphenanthreene) [17]
	*δ* ^1^H-NMR (400 MHz, CD_3_OD)	*δ* ^1^H-NMR (500 MHz, CD_3_OD)
1	6.83 (d, *J* = 2.4 Hz, 1H)	6.90 (1H, d, *J* = 2.4 Hz, H)
2	-	-
3	6.75 (d, *J* = 2.5 Hz, 1H)	6.82 (1H, d, *J* = 2.4 Hz, H)
4	-	-
4a	-	-
4b	-	-
5	9.30 (d, *J* = 9.2 Hz, 1H)	9.37 (1H, d, *J* = 9.3 Hz, 1H)
6	7.07 (dd, *J* = 9.3, 2.8 Hz, 1H)	7.14 (1H, dd, *J* = 2.7, 9.3 Hz, 1H)
7	-	-
8	7.16 (d, *J* = 2.8 Hz, 1H)	7.23 (1H, d, *J* = 2.7 Hz, 1H)
8a	-	-
9	7.48 (d, *J* = 8.8 Hz, 1H)	7.55 (1H, d, *J* = 8.8 Hz, 1H)
10	7.44 (d, *J* = 8.8 Hz, 1H)	7.51 (1H, d, *J* = 8.8 Hz, 1H)
10a		-
OCH_3_	4.02 (br s, 3H)	4.09 (s, 3H)
2, 7-OH	8.54 (br s, 2H),	8.50 (2H, br s, 2-OH and 7-OH)

The constituents of *D. bulbifera* include diterpenoids, glycosides, steroids, steroidal saponins, and sapogenins [19, 20]. To date, only one study has investigated the antibacterial activity of the active components of *D. bulbifera*. This study examined the antibacterial activity of the methanolic extract of *D. bulbifera* and found that the clerodane diterpenoids bafoudiosbulbin A and B demonstrated significant activity against Salmonella species [11]. Phytochemical analysis of *D. bulbifera* revealed a diverse array of chemical constituents, including saponins, tannins, flavonoids, sterols, polyphenols, glycosides, and steroidal saponins. The specific compounds identified can vary based on the geographical origin of the plant, plant part studied, and solvents used for extraction. Notable compounds include flavonol aglycones, such as kaempferol-3, 5-dimethyl ether, caryatin, and catechin, and steroidal sapogenins, such as diosbulbisins and diosbulbisides in plants from China. Bulbs from India contained 8-epidiosbulbin E acetate, whereas those from Cameroon contained clerodane diterpenoids. Additionally, the flowers of *D. bulbifera* from Cameroon contained a broad spectrum of the steroidal saponins dioscoreanosides [21]. Our findings on Flavanthrinin's antibacterial activity align with the broader scientific consensus that flavonoids possess potent antimicrobial properties, capable of inhibiting the growth of various bacterial strains, including those resistant to conventional antibiotics. The structure–activity relationship analysis demonstrated the critical role of specific structural modifications, such as hydroxylation patterns and the presence of functional groups, in enhancing the antibacterial efficacy of flavonoids. This correlation between structural features and antimicrobial potency suggested that Flavanthrinin's effectiveness may be attributed to its unique molecular configuration, which warrants further investigation [22].

### Cytotoxicity assay

**Fig. 5:**
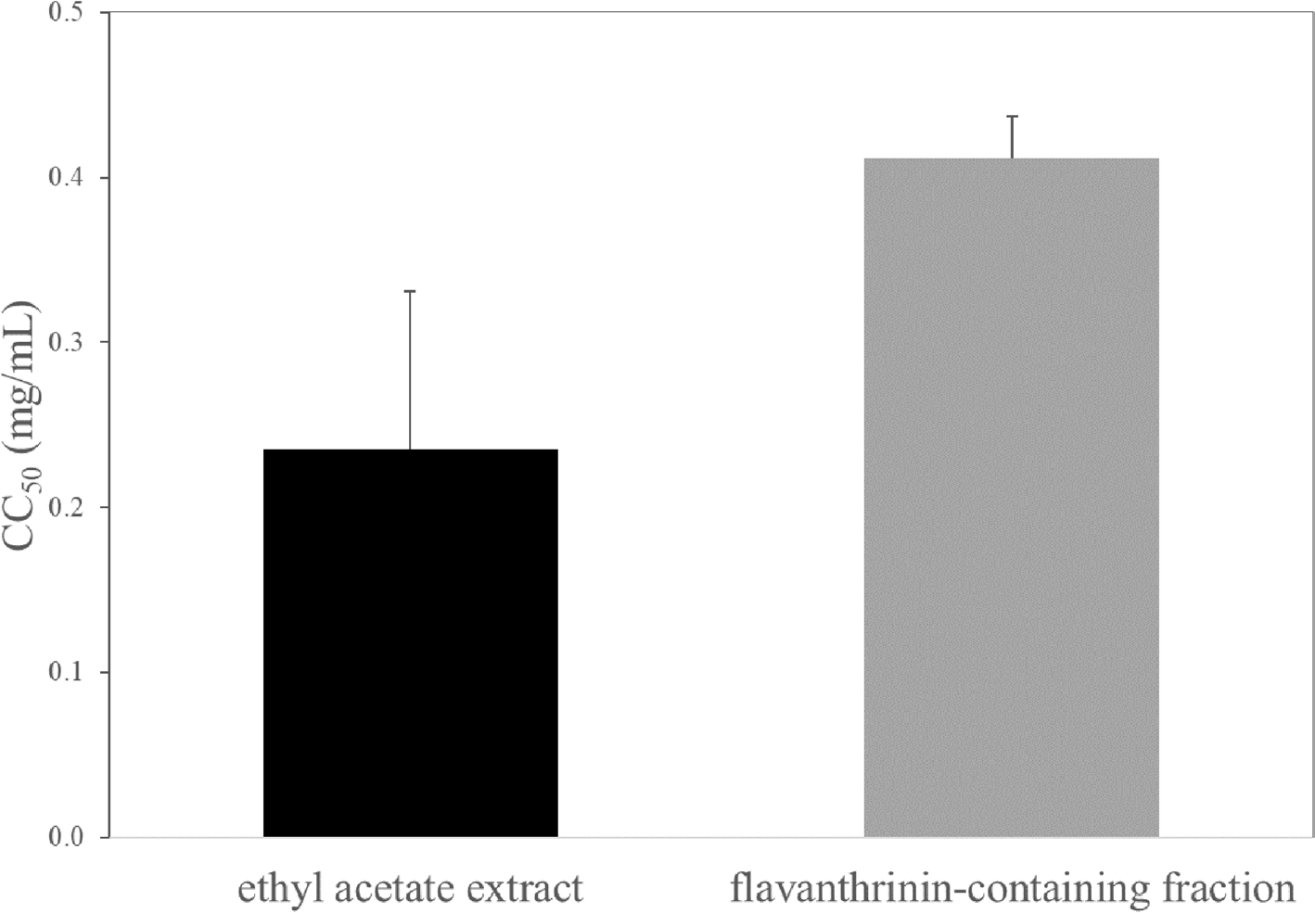
The 50% cytotoxic concentration (CC_50_) of ethyl acetate extract and flavanthrinin-containing fraction

The cytotoxicity of the ethyl acetate extract and the flavanthrinin-containing fraction against Vero cells yielded CC_50_ values of 0.24 ± 0.10 mg/mL and 0.41 ± 0.03 mg/mL, respectively (Fig. 5). Notably, the ethyl acetate extract demonstrated cytotoxicity at concentrations lower than its MIC values (0.78–1.56 mg/mL), indicating potential cellular toxicity at its inhibitory concentrations. Conversely, the flavanthrinin-containing fraction, with a CC_50_ higher than its MIC range (0.04–0.78 mg/mL), suggested a safer profile at effective antibacterial concentrations. This difference underscored the importance of careful consideration of the ethyl acetate extract's cytotoxicity while recognizing the flavanthrinin-containing fraction as a promising candidate for safe antibacterial application.

## Conclusions

Potent antibacterial activity against skin-associated bacteria, including strains of MRSA, was demonstrated by an extract from *D. bulbifera*, particularly due to a component identified as flavanthrinin. Isolation and purification processes, coupled with structural analysis via NMR spectroscopy, offered insights into the active constituents of *D. bulbifera*. Flavanthrinin, demonstrated for its potential as a natural antimicrobial agent due to the antibacterial properties and low cytotoxicity in preliminary assays, suggests a promising potential for *D. bulbifera* extract in managing skin infections. However, comprehensive clinical evaluations are necessary to fully establish the safety and efficacy of such treatments. This research contributes to the exploration of natural sources for antimicrobial agents in the face of increasing antibiotic resistance, emphasizing the need for further study on flavanthrinin and similar compounds.

## Data Availability

All data generated or analysed during this study are included in this published article. Data are available from the corresponding authors upon reasonable request.

## Abbreviations

BHA: Brain heart infusion agar
d: Doublet
dd: Doublet of doublets
m: Multiplet
MHB: Mueller Hinton broth
MIC: Minimum inhibitory concentration
MRSA: Methicillin-resistant *Staphylococcus aureus*
NMR: Nuclear magnetic resonance
PTLC: Preparative thin-layer chromatography
s: Singlet
TLC: Thin-layer chromatography
TMS: Tetramethylsilane
